# Streamlined High-Throughput Data Analysis Workflow for Antibody-Drug Conjugate Biotransformation Characterization

**DOI:** 10.1021/acs.analchem.4c04311

**Published:** 2025-03-11

**Authors:** Kate Liu, Yongling Ai, Hui Yin Tan, Jiaqi Yuan, John K. Meissen, Yuzhuo Zhang, Yue Huang, Anton I. Rosenbaum

**Affiliations:** † Integrated Bioanalysis, Clinical Pharmacology and Safety Sciences, R&D, AstraZeneca, South San Francisco, California 94080, United States; ‡ SCIEX, 1201 Radio Rd., Redwood City, California 94065, United States

## Abstract

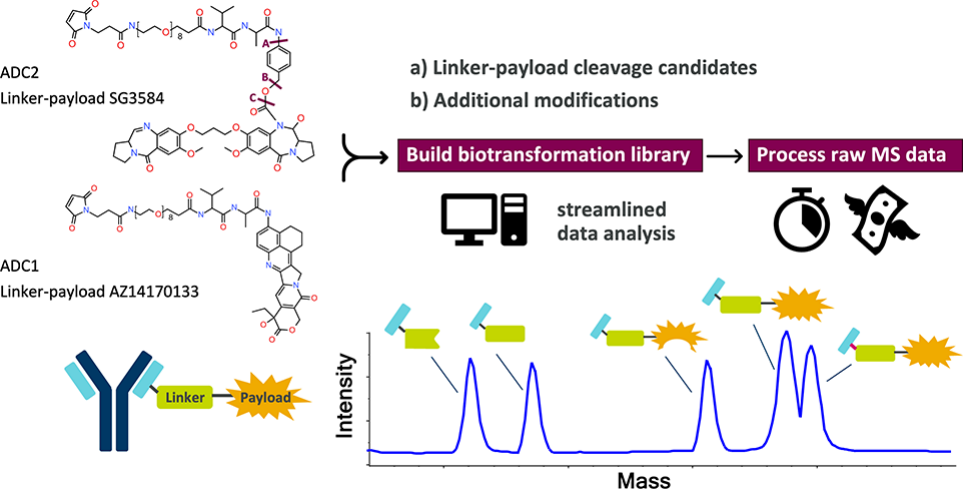

Research into antibody-drug conjugates (ADCs) is currently at an inflection point due to recent clinical impact. ADC biotransformation analysis is key for understanding the structural integrity of ADCs in vivo and is a critical aspect of drug development, especially at the lead selection stage. Data analysis of biotansformed products is hindered by the manual and time-consuming analyte identification process oftentimes taking days to weeks. We developed a streamlined data analysis workflow enabling more automated peak identification using several commercial software tools that significantly improve data processing efficiency. A linker-payload biotransformation library was created for each new molecule and combined with antibody sequence information for peak matching. As a proof of concept, we tested this workflow across different payload and linker types, acquired using different mass spectrometers: an example using a topoisomerase I inhibitor-conjugated ADC (SCIEX ZenoTOF 7600) and a comparison to a published in vivo ADC biotransformation data set for a pyrrolobenzodiazepine-conjugated ADC (ThermoFisher QE HF-X). Using this more automated workflow, we rapidly identified major biotransformation species that were previously found manually including loss of linker-payload, thiosuccinimide ring hydrolysis, cysteinylation at the deconjugation site(s), and partial linker-payload cleavage. This improved data-analysis workflow has demonstrated superb effectiveness in streamlining overall ADC biotransformation identification and enabled quantification that was highly comparable to previously obtained results. Broadening application of advanced analytical techniques to study biotherapeutic biotransformation can now more effectively impact drug development by enabling faster design-test-analyze cycle times, critical in early drug discovery settings, opening new avenues for more effective collaboration between analytical chemists and bioconjugate engineers.

## Introduction

Antibody-drug conjugates (ADCs) as a therapeutic modality has received renewed interest in this decade due to recent clinical successes.^1^ ADC achieves targeted drug delivery by combining potency of cytotoxic drugs with target selectivity of monoclonal antibodies.^2^ The antibody recognizes the target antigen, binds, and is then internalized into cells. The linker provides for controlled release of cytotoxic drugs.

ADCs may undergo structural changes in circulation or in tissues. The antibody portion may experience proteolysis or amino acid modifications such as deamidation and oxidation, which is common to other protein therapeutics.^3^ The linker and payload moieties may undergo changes that directly affect their safety and efficacy. For example, nonspecific payload release may result in undesired toxicity. The released payload components from ADC are subject to small molecule metabolism by enzymes in the liver such as CYP450s.^3^ Typically, conjugated linker-payload remains quite stable in circulation due to limited exposure to metabolizing enzymes. However, biotransformations can still occur on the conjugated linker-payload. One reported example is thiosuccinimide ring hydrolysis commonly observed on cysteine conjugated ADCs. Once thiosuccinimide is hydrolyzed, the linker-payload is no longer subject to elimination through retro-Michael reaction and thus prevents nonspecific deconjugation.^4,5^ Another reported biotransformation is hydrolysis on the payload structure.^6^ Other observed changes to linker-payload are cleavages or partial loss, such as hydrazone cleavage, peptide bond cleavage, carbamate cleavage, disulfide cleavage, etc.^3^ In summary, the biotransformation of conjugated payload can be very different from released payload or small molecules due to inherent distribution differences, enabling exposure to different compartments and enzymes.

Assessing biotransformation of conjugated payload is important for evaluating whether the biotransformed payload component may still be active.^7^ Overall, ADC biotransformation information is valuable for understanding in vivo stability and pharmacokinetics for guiding drug design.

Traditional bioanalysis supporting pharmacokinetic assessments involves monitoring surrogate analytes (peptides or payload) via a targeted bottom-up based approach. With recent advances in high-resolution mass spectrometry (HRMS), we can analyze the macromolecule directly at the intact level without enzyme digestion, revealing biotransformations previously not observed using the traditional LC-MRM (liquid chromatography-multiple reaction monitoring) approach.^8^ In this intact approach, ADCs are enriched from plasma matrix with immunocapture, followed by elution from beads before LC-MS (liquid chromatography–mass spectrometry) analysis. For the drug-to-antibody ratio (DAR) of 8, cysteine-conjugated ADCs used in this study, since interchain disulfide bonds were repurposed as conjugation sites, light chain (LC) and heavy chain (HC) would dissociate in acid elution before analysis on LC-HRMS (liquid chromatography-high resolution mass spectrometry).

**Figure 1:**
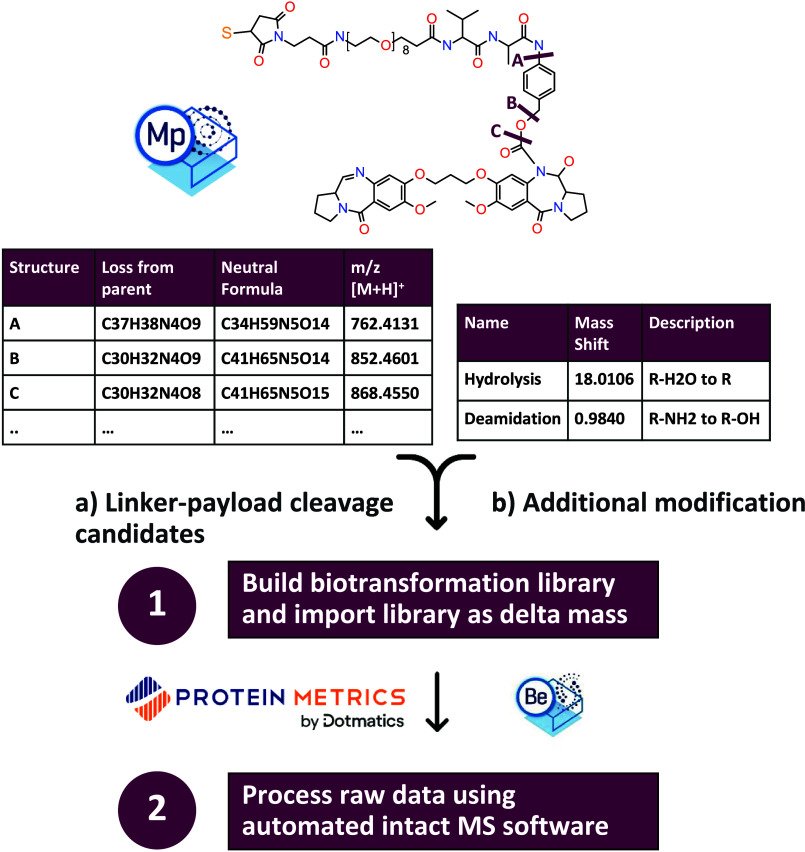
Workflow schematic showing a two-step process where we first build a biotransformation library by combining potential cleavage candidates and additional common potential modifications using Metabolite Pilot (1) and then apply this delta mass library to automated intact data processing software: Protein Metrics and Biologics Explorer (2). SCIEX and Protein Metrics software logos are provided courtesy of SCIEX and Protein Metrics, respectively.

Despite growing interest and more routine evaluation of ADC biotransformation, challenges with data analysis still persist. There is no streamlined software tool for high-throughput mass spectrometry based ADC data annotation.^3^ In contrast, for small molecule biotransformation or metabolism, there are a number of commercial and academic software options available for metabolite prediction and identification.^9^ To bridge the gap, we developed a data analysis workflow that can automatically annotate peaks for ADC biotransformation by integrating a traditional small molecule metabolite prediction tool with large molecule intact MS data analysis software. The general approach of the new workflow has 2 main steps: (1) building a linker-payload biotransformation library by considering truncations and potential modifications, (2) importing the delta mass library to automated intact data processing software (Figure 1). This approach was tested on two historical data sets as proof of concept. With this new approach, the time frame of data analysis shortens from a week (manual search) to just a few hours.

## Experimental Section

### Sample Preparation and LC-MS Analysis for Historical Data

For the first biotransformation case, involving the topoisomerase I inhibitor-conjugated ADC, plasma samples (postdose time points = 1, 24, 72, 120, 168, and 336 h) from cynomolgus monkey (*n* = 1) dosed with ADC1 (a DAR8 ADC containing a topoisomerase I inhibitor payload-AZ14170132) at 15 mg/kg were subjected to immunocapture with SMART IA streptavidin magnetic beads coupled with anti-human Fc and washed extensively with PBS and water. Approximately 1 μg of enriched ADC was eluted with 1% (v/v) formic acid (FA). The eluate was subjected to chromatography separation with a Waters BioResolve RP mAb polyphenyl column (PN186009017; 1% FA, 0.01% TFA in water/ACN as mobile phases) followed by mass spectrometry analysis on a SCIEX 7600 Zeno TOF in full scan mode (MS1 only). Regular calibration ensured the high mass accuracy and resolution necessary for biotransformation analyses.

For the second biotransformation case, involving the pyrrolobenzodiazepine-conjugated ADC, the detailed experimental method was previously described by Huang et al.^8^

### Data Analysis with Biologics Explorer v4.0 (SCIEX, Framingham, MA)

Raw data in .wiff2 format were processed using an intact automated deconvolution workflow in Biologics Explorer (BE). Peak preprocessing was performed prior to deconvolution including spectrum baseline subtraction and noise removal. Deconvolution was performed using a proprietary maximum entropy algorithm with separate retention time windows for light chain and heavy chain elution regions. Peak identification was performed in the Protein Mapping step where the HC and LC amino acid sequences were provided as input. Common fixed modifications included C-terminal lysine loss and N-terminal glutamine to pyroglutamate conversion. Common IgG glycans were selected as possible glycans. For disulfide connections, any remaining unpaired cysteine was allowed to form a cysteinylation. Masses of the original linker-payload and the hydrolyzed (+18 Da) version were entered in the conjugates table. Alternatively, a delta mass library could be uploaded. Identification results after Protein Mapping were manually reviewed.

### Data Analysis with Metabolite Pilot v2.0.4 (SCIEX, Framingham, MA)

To generate the linker-payload biotransformation library, the ADC processing method in Metabolite Pilot (MP) software was used (Molecule Profiler is the updated version of Metabolite Pilot software). The linker-payload structure .mol file was uploaded. The conjugation site was specified at the maleimide ring. Potential compound cleavages were filtered by the site of attachment, meaning considered cleavages were all conjugated to cysteines in the antibody. The maximum number of bonds that can break in the structure was set to 2, and no ring bonds were broken. This generated a list of 46 possible cleavages. For additional biotransformation of the linker-payload, hydrolysis (+H_2_O) and oxidation deamidation to alcohol (R–NH_2_ to R–OH) as well as a combination of the two were added. Combining compound cleavages with biotransformation led to a final exported list of 187 possibilities (Table S1). This list was then reformatted for compatibility with downstream software such as Protein Metrics Byos or BE. Specifically, the name column was reformatted as loss of a chemical formula with or without additional modification (e.g., hydrolysis, deamidation, or combination). The monoisotopic mass (MH+) exported from MP software was converted to a neutral mass.

### Data Analysis with Protein Metrics Byos v5.4 (Dotmatics, Boston, MA)

Raw files were uploaded to the ADC workflow in Protein Metrics software. The custom TIC window was selected to cover LC elution from 1.7 to 2.2 min. The LC sequence was entered, and common IgG modifications (N-terminal Q to pyroGlu and C-terminal K loss) were included. In the delta mass table, the biotransformation library generated from the MP software was imported. In addition, the cysteine adduct was also added as a potential modification. Mass tolerance was set to 3 Da. Deconvolution mass range was from 1000 to 5000 *m*/*z* and 20 000 to 80 000 Da. After analysis, peaks that had multiple assignments within mass tolerance were manually reviewed to select the most plausible assignment based on enzymatic cleavage mechanisms. For quantification, intensities of all peaks were exported and plotted for the fractional abundance of all LC species. Fractional abundance was calculated by each peak intensity divided by the total LC biotransformation peak intensity. Percent difference between new and original values was calculated by subtracting the new from previous values over the mean of the two.

## Results and Discussion

Data analysis for ADC biotransformation characterization has traditionally been a manual and time-consuming process. For a novel ADC molecule, the manual process typically takes longer than a week. The first step is raw data deconvolution from the *m*/*z*-time domain into the mass-time domain using a sliding window. The next step is more laborious, requiring an analytical scientist to manually inspect peaks in deconvoluted spectra at each increment of the retention time window (e.g., 0.5 min) to record all observed peaks above S/N. This mass list would be further refined to keep only peaks found in most PK sample time points. During this process, the identities of these peaks are either matched with biotransformation species reported by literature or proposed manually based on structure and delta mass calculation.

Herein, we describe a new streamlined approach to automatically identify and annotate peaks for high-throughput ADC biotransformation data analysis. This new workflow is demonstrated using two distinct ADC examples.

### Biotransformation Case 1

These data were from cynomolgus monkey plasma samples collected at different time points after dosing with DAR8 cysteine conjugated ADC (ADC1). This ADC appeared to be relatively stable, and only a few biotransformation products were observed, including thio-succinimide ring hydrolysis on the linker and cysteine adduct at the free thiol exposed after payload deconjugation. Other major species observed in the intact data were plasma albumin and the cysteine adduct of albumin.

These data were reprocessed by using the new approach. Because these data did not have linker-payload cleavage, we skipped the first library building step and directly processed raw data using BE. For the protein sequence, we provided HC and LC amino acid sequences. For modifications, we added thio-succinimide hydrolysis as a potential modification to linker-payload. Cysteine adduct was added as a potential modification to any unpaired cysteine. The software performed automated deconvolution, peak picking, and peak annotation using the sequence information and modifications provided. Once parameters were set up, this workflow took less than 30 min to finish.

**Figure 2:**
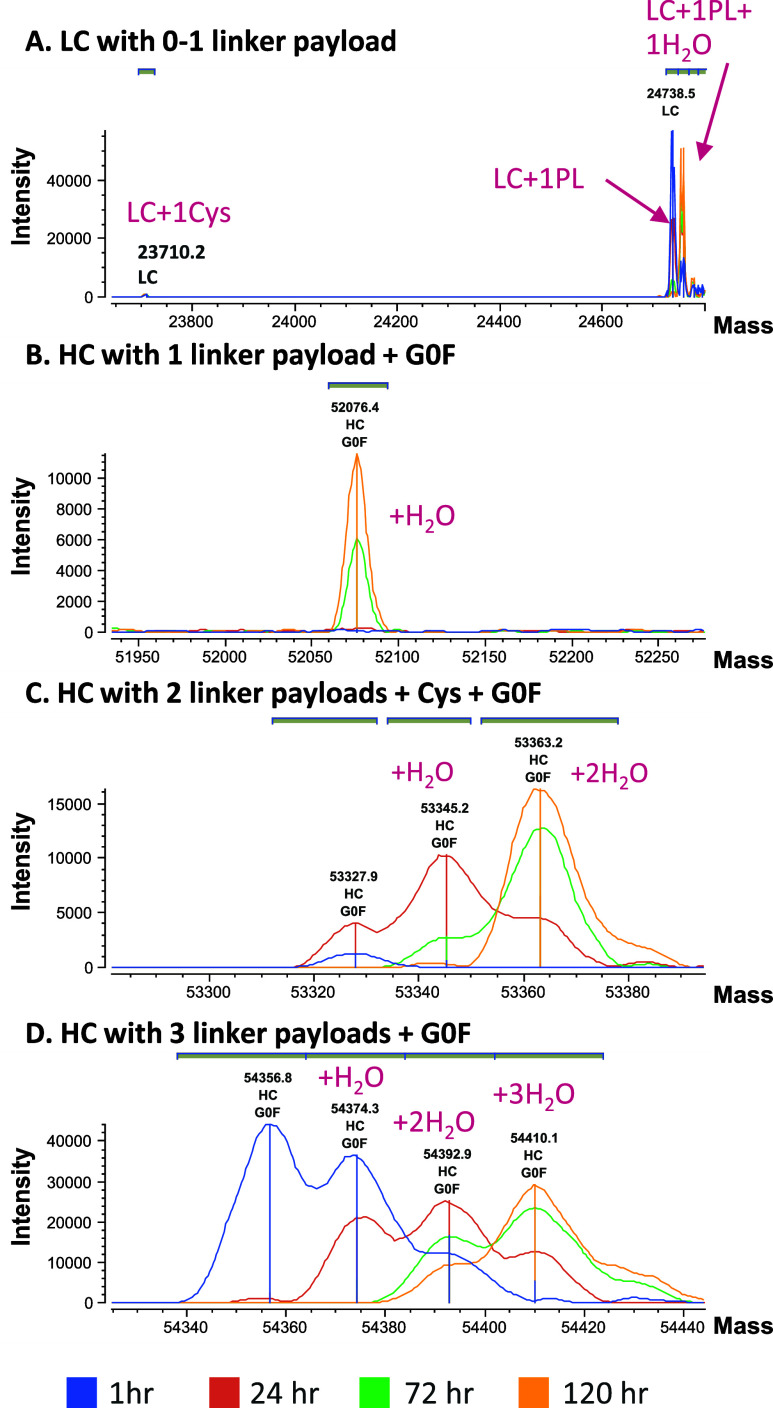
Deconvoluted intact mass spectra showing all major biotransformation peaks associated with ADC1 were identified. (A) LC DAR0-DAR1 peaks. (B) HC DAR1 peak. (C) HC DAR2 peaks with increasing hydrolysis over time. (D) HC DAR3 peaks with increasing hydrolysis over time.

Using this new workflow, we were able to identify all major biotransformation species previously found by using the time-consuming manual process (Table 1 and Figure 2). Hydrolysis was the major biotransformation in LC and HC. For HC, we saw a gradual increase in thio-succinimide hydrolysis over time, resulting in complete hydrolysis at 120 h postdose. The other biotransformation was cysteine adduct formation once free thiol was exposed from linker-payload deconjugation. Interestingly, for HC, only DAR2 species forming the cysteine adduct at the free thiol was observed. We previously hypothesized that the reason we did not observe cysteine adducts in HC DAR1 is because, if the 2 free thiol sites are spatially close, they could form an intrachain disulfide bond^4^ or the abundance of this species was below the detection limit of this method. Albumin species were not automatically identified because we provided only the antibody sequence to the software. However, we expect this approach to also work for the identification of biotransformation products where the payload has been transferred to endogenous proteins, such as albumin.

**Table 1:** Major Biotransformation Species Identified Using the SCIEX Biologics Explorer^a^

Theoretical Mass (Da)	RT (min)	Proposed ID	Ab Chain	DAR (# payloads)	Mass Accuracy (ppm)	Identified in BE
23710	3.35	LC + 1Cys	LC	0	0.2	Yes
24739	3.61	LC + 1PL	LC	1	–40	Yes
24758	3.66	LC + 1PL + 1H_2_O	LC	1	–6	Yes
52076	4.22	HC + 1PL + 1H_2_O + G0F	HC	1	7	Yes
53325	4.56	HC + 2PL + 1Cys + G0F	HC	2	53	Yes
53343	4.56	HC + 2PL + 1Cys + 1H_2_O + G0F	HC	2	33	Yes
53363	4.39	HC + 2PL + 1Cys + 2H_2_O + G0F	HC	2	29	Yes
54354	4.88	HC + 3PL + G0F	HC	3	47	Yes
54373	4.81	HC + 3PL + 1H_2_O + G0F	HC	3	32	Yes
54391	4.81	HC + 3PL + 2H_2_O + G0F	HC	3	39	Yes
54409	4.74	HC + 3PL + 3H_2_O + G0F	HC	3	18	Yes
65945	4.22	Albumin	N/A	N/A	N/A	N/A
66063	4.22	Albumin + 1Cys	N/A	N/A	N/A	N/A

a PL indicates payload-linker.

### Biotransformation Case 2

In this study, Sprague–Dawley rats were dosed with DAR8 cysteine conjugated ADC, trastuzumab with a pyrrolobenzodiazepine (PBD)-dimer payload (SG3584). The data were acquired on a QE HF-X instrument (Thermo Fisher Scientific, Waltham, MA). The data were previously deconvoluted using BioPharma Finder, and peaks were manually annotated as described previously.^8^ For this ADC, we observed expected biotransformation such as thio-succinimide hydrolysis but also unexpected peaks that were attributed to sequential linker-payload degradation.

In the new workflow, we first created a biotransformation library for the SG3584 linker-payload in MP software. We uploaded the structure of linker-payload and selected up to 2 bond cleavages excluding breaking ring bonds (Figure S1). We also selected additional modifications including hydrolysis and deamidation as well as the two combined from the software built-in biotransformation set. This entire list of delta masses was imported to Protein Metrics Byos for peak matching (Table S1).

**Figure 3:**
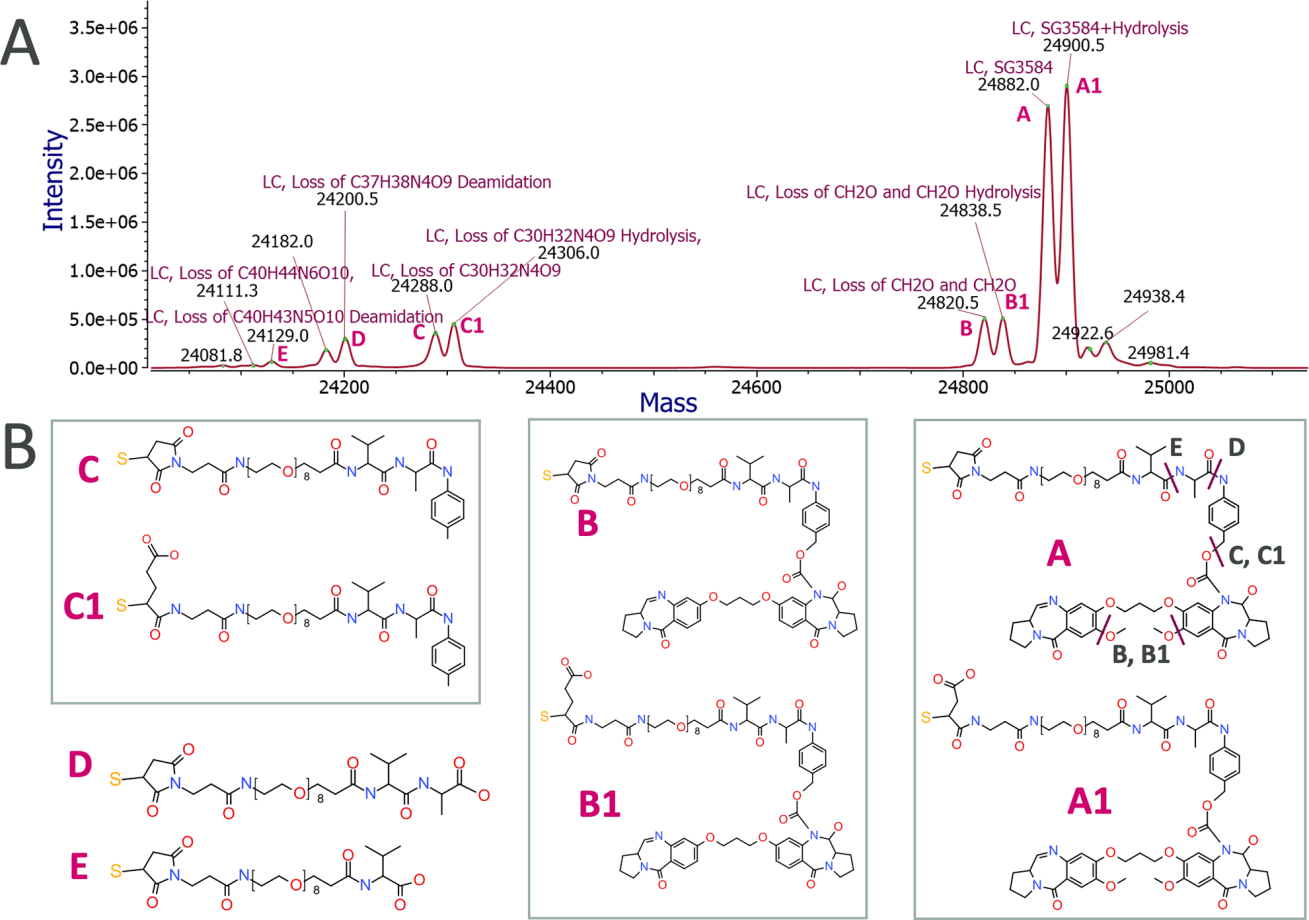
Light chain (LC)-SG3584 biotransformation species identified using the new workflow demonstrated by an example data from the 24 h time point. (A) Annotated mass spectrum showing all major LC biotransformation peaks. (B) Proposed structures for biotransformation species. Structure A was the parent linker-payload, and structure A1 was the thiosuccinimide hydrolyzed version. Structures B and B1 were missing two O-methyl groups on the payload. Structures C and C1 had a complete loss of payload. Structures D and E had a further loss of the linker and may have originated from A, B, or C. Structures in B are reproduced in part from ref (8). Available under a CC-BY 4.0 license. Copyright 2021 The Authors.

The new combined workflow utilizing MP and Byos was able to annotate all of the peaks that were proposed in the original publication (Figure 3). We observed the full linker-payload (A, A1) as well as partial loss (B, B1, C, C1, D, E). Structure A1 is the thiosuccinimide hydrolyzed version of A. Proposed structures B, B1 and C, C1 were likely impurities from payload synthesis and not strictly biotransformation species since they were observed at very early time points (2 min postdose). Proposed structures D and E were further cleaved along the linker-amide bond. All proposed structures based on intact mass would require additional experiments such as MS^2^ confirmation using collision-induced dissociation (CID) or electron activated dissociation (EAD) to fully confirm their identity.^10^ Input from protein engineering/medicinal chemistry or bioconjugation engineers can further assist in the identification of known impurities in the ADC reference material to differentiate from in vivo formed biotransformations. Mass accuracies for proposed structures A–E and other matched species within mass tolerance are listed in Table S2. When there were multiple matched results for a peak, judgment calls were made to select the most plausible identification based on known enzyme cleavage possibilities and payload impurities. Other scenarios to consider were any known modifications on the mAb portion of the ADC.

High mass accuracy and resolution are clearly important in making such judgment calls, and high quality data generated from high resolution-accuracy mass spectrometry (HRAMS) instruments with more concentrated samples can improve data quality. QTOF instruments can typically achieve 50 ppm or better mass accuracy for heavy chains (50 kDa, with ±2.5 Da). This window is sufficient for the identification of most common modifications, except deamidation (0.98 Da). On Orbitrap systems, mass resolution is typically a setting that you can choose in the acquisition method while balancing resolution and sensitivity at a high mass range. The actual achievable/effective mass accuracy on any HRAMS instrument depends on several factors such as calibration, analyte concentration, buffer used in the system, and spectral complexity.

Although Protein Metrics Byos was used in this example, other commercial mass spectrometry software, such as BE or BioPharma Finder, should be able to incorporate the delta mass library for automated peak identification as well, making this method widely applicable.

**Figure 4:**
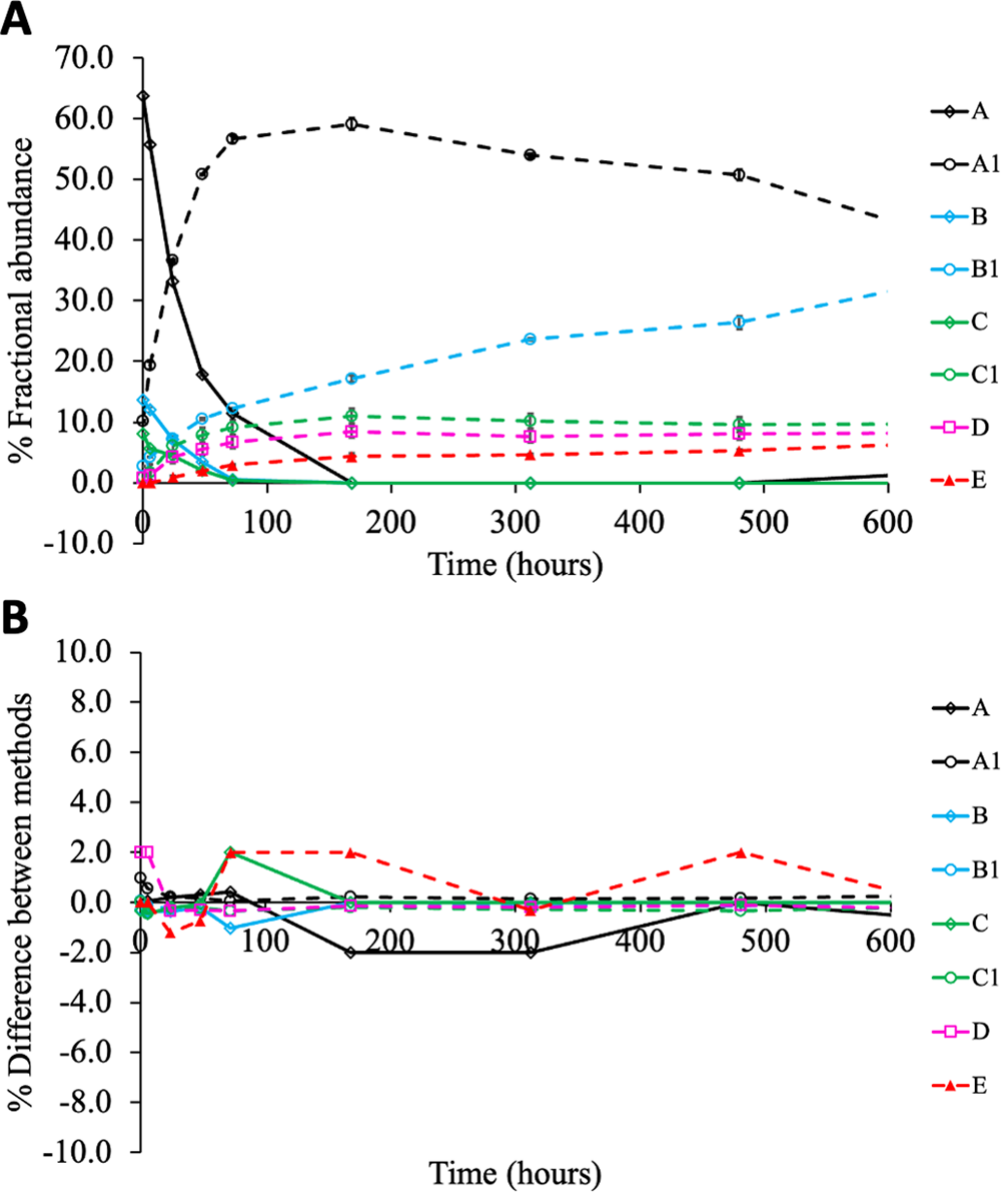
Fractional abundance of major LC biotransformation species over 28 days after administration of ADC in rats (*n* = 3). (A) Quantification results using the new workflow. Data points with no observed signal intensity were plotted as zero. Error bars represent standard deviation of biological replicates. (B) Percent difference between new and original (from ref (8)) fractional abundance values. This percent difference was calculated by subtracting new from previous fractional abundance over the mean of the two values.

To compare quantification between new and previous workflows, we plotted the change in major metabolites in LC as percent fractional abundance, which was calculated from peak intensity over total peak intensity at each time point for each animal (Figure 4A). The new data analysis workflow successfully recapitulated the overall trend in the previous publication.^8^ The minor % difference between newly obtained and published values (Figure 4B) could be attributed to differences in the deconvolution algorithm and the parameters used in BioPharma Finder vs Byos software.

## Conclusion

This work showcases how ADC biotransformation can be routinely and rapidly characterized by using commercial software tools. Interest in ADC biotransformation in the analytical community has been increasing, as the number of ADC candidates continues to grow. However, the lack of streamlined software and analytical processes for high-resolution-accurate mass data has been impeding wide application of biotransformation analysis to effectively impact drug development. In this study, we used two ADC examples with structurally different linker-payloads and data acquired using two different high resolution instrument platforms to demonstrate a novel, more streamlined, data processing workflow. Combining linker-payload structural cleavage prediction and additional common biotransformations, we can apply automated peak matching and rapidly propose new biotransformation structures. This high-throughput workflow is particularly beneficial for early drug discovery, where biotransformation insights can guide lead selection through iterative design. However, it requires *a priori* knowledge/prediction of potential biotransformation pathways for ADCs.

## Supporting Information Available

Processing parameters in MP, biotransformation library, and matched peaks results for proposed structures in Case 2 (PDF)
