# Supplementary material for: Streamlined High-Throughput Data Analysis Workflow for Antibody-Drug Conjugate Biotransformation Characterization
Source: Anal Chem. 2025 Mar 11;97(11):5919–25. doi: 10.1021/acs.analchem.4c04311 (PMC11948178; doi:10.1021/acs.analchem.4c04311)
Supplement: Supplementary file 1 — ac4c04311_si_001.pdf [file ac4c04311_si_001.pdf]

Supporting Information

Streamlined High-Throughput Data Analysis Workflow for ADC Biotransformation Characterization

Kate Liu[1], Yongling Ai[1], Hui Yin Tan[1], Jiaqi Yuan[1], John K. Meissen[1], Yuzhuo Zhang[2], Yue Huang[1†], Anton I. Rosenbaum[1*††]

1. Integrated Bioanalysis, Clinical Pharmacology and Safety Sciences, R&D, AstraZeneca, South San Francisco, CA 94080, USA

2. SCIEX, 1201 Radio Rd, Redwood City, CA 94065, USA

Corresponding Author

*Anton I. Rosenbaum - 121 Oyster Point Blvd, South San Francisco, CA 94080, USA; Tel: +1-650-379-3099; anton.rosenbaum.phd@gmail.com

Present Addresses

†Yue Huang – Revolution Medicines, 700 Saginaw Drive, Redwood City, CA 94063, USA; Tel: +1-650-481-6801; E-mail: yhuang@revmed.com

††Anton Rosenbaum– Vera Therapeutics, 2000 Sierra Point Parkway, Brisbane, CA 94005, USA; anton.rosenbaum@veratx.com

Table of Contents:

Figure S1. Processing parameters in SCIEX Metabolite Pilot used in Case 2 S3

Table S1. Biotransformation library used in Case 2 S4

Table S2. Peak Matching Results from Protein Metrics Byos used in Case 2 S9

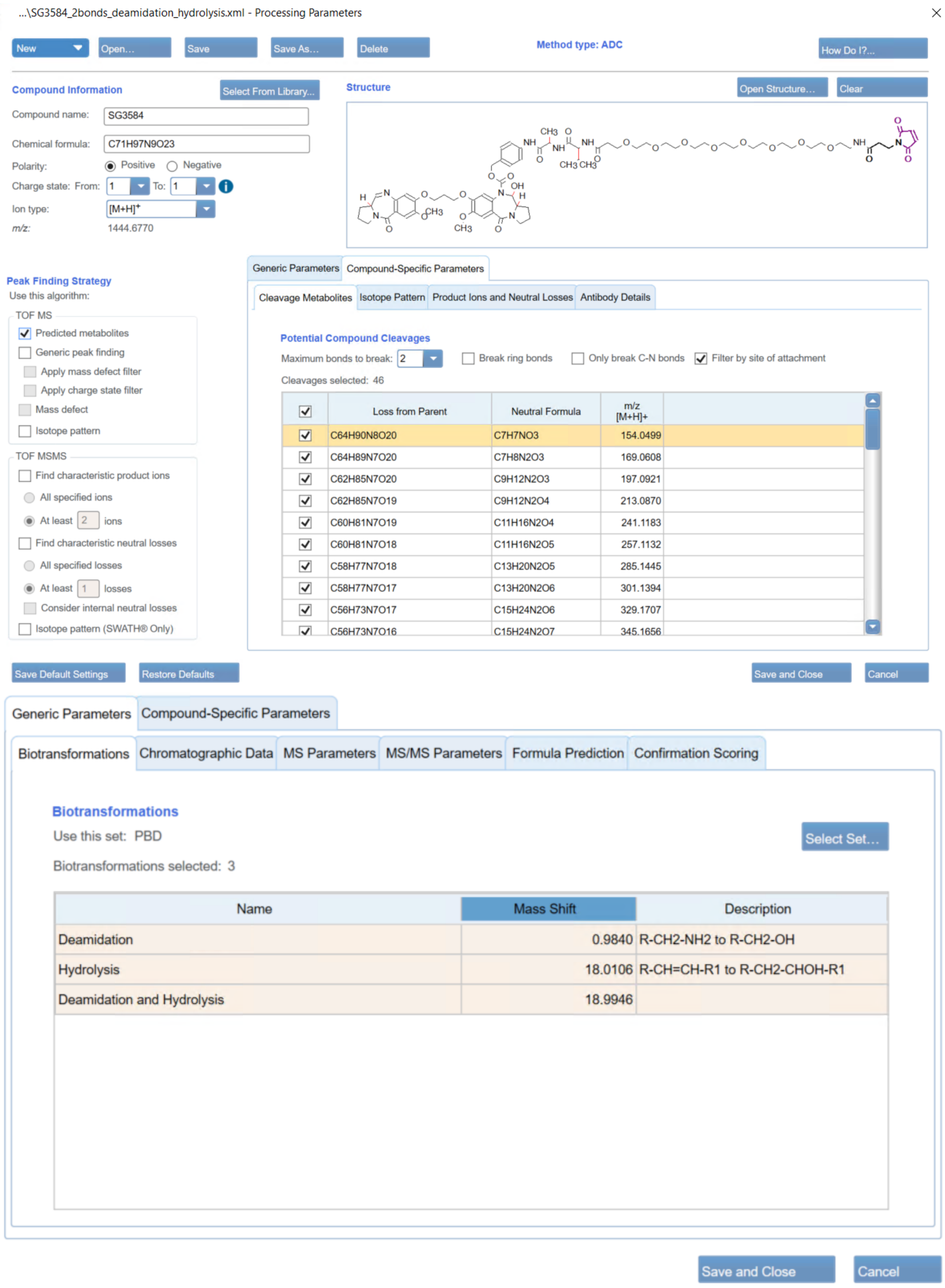

Figure S1. Processing parameters in SCIEX Metabolite Pilot showing settings used to generate biotransformation library. For linker-payload structural cleavages, we allowed up to 2 bond cleavages and no breaking of ring bonds. This generated a list of 46 bond cleavages initially. Additional biotransformation including hydrolysis, deamidation, as well as combination of the two were also added. Combining bond cleavages with additional modification led to a total exported list of 187 possibilities.

Modification name	Delta Mass (Da)
SG3584	1443.6796
SG3584 Deamidation	1444.653
SG3584 Hydrolysis	1461.6796
SG3584 Deamidation and Hydrolysis	1462.6636
Loss of CH2	1429.6534
Loss of CH2 Deamidation	1430.6374
Loss of CH2 Hydrolysis	1447.664
Loss of CH2 Deamidation and Hydrolysis	1448.648
Loss of O	1427.6741
Loss of O Deamidation	1428.6581
Loss of O Hydrolysis	1445.6847
Loss of O Deamidation and Hydrolysis	1446.6687
Loss of CH2 and CH2	1415.6377
Loss of CH2 and CH2 Deamidation	1416.6217
Loss of CH2 and CH2 Hydrolysis	1433.6483
Loss of CH2 and CH2 Deamidation and Hydrolysis	1434.6323
Loss of CH2O	1413.6584
Loss of CH2O Deamidation	1414.6424
Loss of CH2O Hydrolysis	1431.669
Loss of CH2O Deamidation and Hydrolysis	1432.653
Loss of CH2 and CH2O	1399.6428
Loss of CH2 and CH2O Deamidation	1400.6268
Loss of CH2 and CH2O Hydrolysis	1417.6534
Loss of CH2 and CH2O Deamidation and Hydrolysis	1418.6374
Loss of CH2O and O	1397.6635
Loss of CH2O and O Deamidation	1398.6475
Loss of CH2O and O Hydrolysis	1415.6741
Loss of CH2O and O Deamidation and Hydrolysis	1416.6581
Loss of CH2O and CH2O	1383.6479
Loss of CH2O and CH2O Deamidation	1384.6319
Loss of CH2O and CH2O Hydrolysis	1401.6585
Loss of CH2O and CH2O Deamidation and Hydrolysis	1402.6425
Loss of C13H12N2O2	1215.5791
Loss of C13H12N2O2 Deamidation	1216.5631
Loss of C13H12N2O2 Hydrolysis	1233.5897
Loss of C13H12N2O2 Deamidation and Hydrolysis	1234.5737
Loss of C13H12N2O2 and CH2	1201.5635
Loss of C13H12N2O2 and CH2 Deamidation	1202.5475
Loss of C13H12N2O2 and CH2 Hydrolysis	1219.5741
Loss of C13H12N2O2 and CH2 Deamidation and Hydrolysis	1220.5581

Loss of C13H12N2O3	1199.5842
Loss of C13H12N2O3 Deamidation	1200.5682
Loss of C13H12N2O3 Hydrolysis	1217.5948
Loss of C13H12N2O3 Deamidation and Hydrolysis	1218.5788
Loss of C13H12N2O3 and CH2	1185.5686
Loss of C13H12N2O3 and CH2 Deamidation	1186.5526
Loss of C13H12N2O3 and CH2 Hydrolysis	1203.5792
Loss of C13H12N2O3 and CH2 Deamidation and Hydrolysis	1204.5632
Loss of C13H12N2O3 and O	1183.5893
Loss of C13H12N2O3 and O Deamidation	1184.5733
Loss of C13H12N2O3 and O Hydrolysis	1201.5999
Loss of C13H12N2O3 and O Deamidation and Hydrolysis	1202.5839
Loss of C13H12N2O3 and CH2O	1169.5737
Loss of C13H12N2O3 and CH2O Deamidation	1170.5577
Loss of C13H12N2O3 and CH2O Hydrolysis	1187.5843
Loss of C13H12N2O3 and CH2O Deamidation and Hydrolysis	1188.5683
Loss of C16H18N2O3	1157.5373
Loss of C16H18N2O3 Deamidation	1158.5213
Loss of C16H18N2O3 Hydrolysis	1175.5479
Loss of C16H18N2O3 Deamidation and Hydrolysis	1176.5319
Loss of C16H18N2O3 and CH2	1143.5216
Loss of C16H18N2O3 and CH2 Deamidation	1144.5056
Loss of C16H18N2O3 and CH2 Hydrolysis	1161.5322
Loss of C16H18N2O3 and CH2 Deamidation and Hydrolysis	1162.5162
Loss of C16H18N2O4	1141.5424
Loss of C16H18N2O4 Deamidation	1142.5264
Loss of C16H18N2O4 Hydrolysis	1159.553
Loss of C16H18N2O4 Deamidation and Hydrolysis	1160.537
Loss of C16H18N2O4 and CH2	1127.5267
Loss of C16H18N2O4 and CH2 Deamidation	1128.5107
Loss of C16H18N2O4 and CH2 Hydrolysis	1145.5373
Loss of C16H18N2O4 and CH2 Deamidation and Hydrolysis	1146.5213
Loss of C16H18N2O4 and O	1125.5474
Loss of C16H18N2O4 and O Deamidation	1126.5314
Loss of C16H18N2O4 and O Hydrolysis	1143.558
Loss of C16H18N2O4 and O Deamidation and Hydrolysis	1144.542
Loss of C16H18N2O4 and CH2O	1111.5318
Loss of C16H18N2O4 and CH2O Deamidation	1112.5158

Loss of C16H18N2O4 and CH2O Hydrolysis	1129.5424
Loss of C16H18N2O4 and CH2O Deamidation and Hydrolysis	1130.5264
Loss of C29H32N4O7	895.4419
Loss of C29H32N4O7 Deamidation	896.4259
Loss of C29H32N4O7 Hydrolysis	913.4525
Loss of C29H32N4O7 Deamidation and Hydrolysis	914.4365
Loss of C30H32N4O8	867.447
Loss of C30H32N4O8 Deamidation	868.431
Loss of C30H32N4O8 Hydrolysis	885.4576
Loss of C30H32N4O8 Deamidation and Hydrolysis	886.4416
Loss of C30H32N4O9	851.4521
Loss of C30H32N4O9 Deamidation	852.4361
Loss of C30H32N4O9 Hydrolysis	869.4627
Loss of C30H32N4O9 Deamidation and Hydrolysis	870.4467
Loss of C37H38N4O9	761.4051
Loss of C37H38N4O9 Deamidation	762.3891
Loss of C37H38N4O9 Hydrolysis	779.4157
Loss of C37H38N4O9 Deamidation and Hydrolysis	780.3997
Loss of C37H39N5O9	746.3942
Loss of C37H39N5O9 Deamidation	747.3782
Loss of C37H39N5O9 Hydrolysis	764.4048
Loss of C37H39N5O9 Deamidation and Hydrolysis	765.3888
Loss of C40H43N5O10	690.368
Loss of C40H43N5O10 Deamidation	691.352
Loss of C40H43N5O10 Hydrolysis	708.3786
Loss of C40H43N5O10 Deamidation and Hydrolysis	709.3626
Loss of C40H44N6O10	675.3571
Loss of C40H44N6O10 Deamidation	676.3411
Loss of C40H44N6O10 Hydrolysis	693.3677
Loss of C40H44N6O10 Deamidation and Hydrolysis	694.3517
Loss of C45H52N6O11	591.2996
Loss of C45H52N6O11 Deamidation	592.2836
Loss of C45H52N6O11 Hydrolysis	609.3102
Loss of C45H52N6O11 Deamidation and Hydrolysis	610.2942
Loss of C45H53N7O11	576.2887
Loss of C45H53N7O11 Deamidation	577.2727
Loss of C45H53N7O11 Hydrolysis	594.2993
Loss of C45H53N7O11 Deamidation and Hydrolysis	595.2833
Loss of C48H57N7O12	520.2625
Loss of C48H57N7O12 Deamidation	521.2465
Loss of C48H57N7O12 Hydrolysis	538.2731
Loss of C48H57N7O12 Deamidation and Hydrolysis	539.2571

Loss of C48H57N7O13	504.2676
Loss of C48H57N7O13 Deamidation	505.2516
Loss of C48H57N7O13 Hydrolysis	522.2782
Loss of C48H57N7O13 Deamidation and Hydrolysis	523.2622
Loss of C50H61N7O13	476.2363
Loss of C50H61N7O13 Deamidation	477.2203
Loss of C50H61N7O13 Hydrolysis	494.2469
Loss of C50H61N7O13 Deamidation and Hydrolysis	495.2309
Loss of C50H61N7O14	460.2414
Loss of C50H61N7O14 Deamidation	461.2254
Loss of C50H61N7O14 Hydrolysis	478.252
Loss of C50H61N7O14 Deamidation and Hydrolysis	479.236
Loss of C52H65N7O14	432.2101
Loss of C52H65N7O14 Deamidation	433.1941
Loss of C52H65N7O14 Hydrolysis	450.2207
Loss of C52H65N7O14 Deamidation and Hydrolysis	451.2047
Loss of C52H65N7O15	416.2151
Loss of C52H65N7O15 Deamidation	417.1991
Loss of C52H65N7O15 Hydrolysis	434.2257
Loss of C52H65N7O15 Deamidation and Hydrolysis	435.2097
Loss of C54H69N7O15	388.1838
Loss of C54H69N7O15 Deamidation	389.1678
Loss of C54H69N7O15 Hydrolysis	406.1944
Loss of C54H69N7O15 Deamidation and Hydrolysis	407.1784
Loss of C54H69N7O16	372.1889
Loss of C54H69N7O16 Deamidation	373.1729
Loss of C54H69N7O16 Hydrolysis	390.1995
Loss of C54H69N7O16 Deamidation and Hydrolysis	391.1835
Loss of C56H73N7O16	344.1576
Loss of C56H73N7O16 Deamidation	345.1416
Loss of C56H73N7O16 Hydrolysis	362.1682
Loss of C56H73N7O16 Deamidation and Hydrolysis	363.1522
Loss of C56H73N7O17	328.1627
Loss of C56H73N7O17 Deamidation	329.1467
Loss of C56H73N7O17 Hydrolysis	346.1733
Loss of C56H73N7O17 Deamidation and Hydrolysis	347.1573
Loss of C58H77N7O17	300.1314
Loss of C58H77N7O17 Deamidation	301.1154
Loss of C58H77N7O17 Hydrolysis	318.142
Loss of C58H77N7O17 Deamidation and Hydrolysis	319.126
Loss of C58H77N7O18	284.1365
Loss of C58H77N7O18 Deamidation	285.1205

Loss of C58H77N7O18 Hydrolysis	302.1471
Loss of C58H77N7O18 Deamidation and Hydrolysis	303.1311
Loss of C60H81N7O18	256.1052
Loss of C60H81N7O18 Deamidation	257.0892
Loss of C60H81N7O18 Hydrolysis	274.1158
Loss of C60H81N7O18 Deamidation and Hydrolysis	275.0998
Loss of C60H81N7O19	240.1103
Loss of C60H81N7O19 Deamidation	241.0943
Loss of C60H81N7O19 Hydrolysis	258.1209
Loss of C60H81N7O19 Deamidation and Hydrolysis	259.1049
Loss of C62H85N7O19	212.079
Loss of C62H85N7O19 Deamidation	213.063
Loss of C62H85N7O19 Hydrolysis	230.0896
Loss of C62H85N7O19 Deamidation and Hydrolysis	231.0736
Loss of C62H85N7O20	196.0841
Loss of C62H85N7O20 Deamidation	197.0681
Loss of C62H85N7O20 Hydrolysis	214.0947
Loss of C62H85N7O20 Deamidation and Hydrolysis	215.0787
Loss of C64H89N7O20	168.0528
Loss of C64H89N7O20 Deamidation	169.0368
Loss of C64H89N7O20 Hydrolysis	186.0634
Loss of C64H89N7O20 Deamidation and Hydrolysis	187.0474
Loss of C64H90N8O20	153.0419
Loss of C64H90N8O20 Deamidation	154.0259
Loss of C64H90N8O20 Hydrolysis	171.0525
Loss of C64H90N8O20 Deamidation and Hydrolysis	172.0365

Table S1. Biotransformation library exported from SCIEX Metabolite Pilot and then reformatted for Protein Metrics Byos upload as a delta mass table.

Labeled Structure	Peak Mass Observed	Matched IDs (can be multiple for a peak)	Delta Mass from Calc.
A1	24900.5	**LC, SG3584+Hydrolysis**	-0.07
		LC, SG3584+Deamidation and Hydrolysis	-1.06
A	24882.0	**LC, SG3584**	-0.61
		LC, SG3584+Deamidation	-1.58
		LC, Loss of O Hydrolysis	-2.61
B1	24838.5	**LC, Loss of CH2O and CH2O Hydrolysis**	-2.04
		LC, Loss of CH2 and CH2O	-0.02
		LC, Loss of CH2 and CH2O Deamidation	-1.01
		LC, Loss of CH2O and O	1.95
		LC, Loss of CH2O and O Deamidation	0.97
B	24820.5	**LC, Loss of CH2O and CH2O**	-2.09
C1	24306.0	**LC, Loss of C30H32N4O9 Hydrolysis**	-2.38
		LC, Loss of C30H32N4O8	-0.36
		LC, Loss of C30H32N4O8 Deamidation	-1.35
C	24288.0	**LC, Loss of C30H32N4O9**	-2.39
D	24200.5	**LC, Loss of C37H38N4O9 Deamidation**	-0.76
		LC, Loss of C37H38N4O9	0.23
		LC, Loss of C37H39N5O9 Hydrolysis	-2.77
E	24129.0	**LC, Loss of C40H43N5O10 Deamidation**	-1.25
		LC, Loss of C40H43N5O10	-0.26

Table S2. Peak Matching Results from Protein Metrics Byos for proposed structures. When multiple matches were possible within the mass tolerance (3 Da), scientific judgement calls were made based on chemical structure and known enzymatic cleavages. The proposed ID for each peak was highlighted in bold.
